# Impact of Remote Symptom Management on Exercise Adherence After Video-Assisted Thoracic Surgery for Lung Cancer in a Tertiary Hospital in China: Protocol for a Prospective Randomized Controlled Trial

**DOI:** 10.2196/60420

**Published:** 2025-01-01

**Authors:** Jianwei Su, Cuiling Ye, Qian Zhang, Yi Liang, Jianwei Wu, Guixi Liang, Yalan Cheng, Xiaojuan Yang

**Affiliations:** 1 Department of cardiothoracic Surgery Zhongshan City People’s Hospital Zhongshan China

**Keywords:** thoracic surgery, rehabilitation medicine, patient-reported outcome measures, patient participation, telemedicine, eHealth, mobile phone

## Abstract

**Background:**

Regular pulmonary rehabilitation exercises are crucial for patients with lung cancer after surgery. However, poor adherence to outpatient exercises is difficult to address due to inadequate supervision. The integration of remote symptom management through electronic patient-reported outcomes (ePROs) offers a potential solution to improve adherence by enabling more effective monitoring and intervention.

**Objective:**

This study aims to evaluate the impact of ePRO-based remote symptom management on enhancing adherence to outpatient pulmonary rehabilitation exercises following video-assisted thoracic surgery for lung cancer.

**Methods:**

In this single-center, prospective, randomized controlled trial, 736 patients undergoing minimally invasive lung resection will be recruited. All patients will use a smartphone app for perioperative management, allowing periodic PRO measurement and recording of exercise participation. Upon discharge, patients will be randomly assigned 1:1 into either an intervention or control group. The intervention group will complete the Perioperative Symptom Assessment for Patients Undergoing Lung Surgery (PSA-Lung) scale on the day of discharge and postdischarge days 3, 7, 14, 21, and 28. Alerts will be triggered at the provider side if any of the 5 core symptoms (pain, cough, shortness of breath, sleep disturbance, and fatigue) scored ≥4, prompting remote symptom management. The control group will complete the PRO measures without triggering alerts. The primary outcome is the rehabilitation exercise adherence rate. Secondary outcomes include postdischarge pulmonary complication rate, 30-day readmission rate, trajectory of symptom severity changes, exercise participation rate, and patient satisfaction.

**Results:**

The enrollment of study participants started in December 2023 and is expected to end in March 2025. The final comprehensive analysis of the results is planned for May 2025, after all data have been collected and thoroughly reviewed.

**Conclusions:**

This study is among the first to investigate the feasibility and effectiveness of ePRO-based remote symptom management in enhancing rehabilitation adherence after video-assisted thoracic surgery for lung cancer. If successful, this approach could significantly influence postoperative care practices and potentially be adopted in similar settings.

**Trial Registration:**

ClinicalTrials.gov NCT05990946; https://clinicaltrials.gov/study/NCT05990946

**International Registered Report Identifier (IRRID):**

DERR1-10.2196/60420

## Introduction

Lung cancer remains a predominant cause of cancer-related morbidity and mortality worldwide. Specifically in China, the incidence rate reached 828,000 cases in 2016 [1]. Non–small cell lung cancer is the most prevalent subtype. With the increasing adoption of lung cancer screening, more patients are being diagnosed at early stages. Surgical resection is the gold standard for treating early-stage, non–small cell lung cancer, and the paradigm of enhanced recovery after surgery has been widely adopted in perioperative care [2].

While enhanced recovery after surgery effectively shortens inpatient stays, its benefits are often offset by inadequate outpatient management stemming from limited resources, which can adversely affect clinical outcomes and postdischarge quality of life [3]. One critical factor in postoperative recovery is adherence to pulmonary rehabilitation exercises. However, adherence rates are suboptimal due to insufficient supervision and guidance.

Following discharge, many patients face decreased exercise adherence rates, ranging from 50% to 70% according to studies focusing on patients with musculoskeletal disorders [4-6]. Several factors influence adherence, including self-efficacy, personal beliefs, sense of self-control, physical and psychological condition, clinical symptoms like pain, and perceived forgetfulness [7-11].

Moreover, many patients continue to experience symptoms such as coughing, pain, poor sleep, and breathlessness after discharge, which may substantially undermine their exercise adherence [12]. In the era of patient-centered care, remote symptom management through electronic patient-reported outcomes (ePROs) is emerging as a promising approach to improve outpatient quality of life and reduce postoperative complications [13-16]. By providing effective strategies for timely monitoring and managing patients’ symptoms, ePRO-based interventions may help patients overcome barriers to exercise and enhance their self-efficacy and motivation, thus promoting exercise adherence [17].

There is a pressing need for research to investigate the impact of postoperative symptoms on exercise adherence and to evaluate whether ePRO-based remote symptom management can effectively improve adherence to outpatient rehabilitation exercises [18,19].

Therefore, this study aims to assess the feasibility and preliminary effects of remote symptom management based on ePROs on enhancing adherence to outpatient rehabilitation exercises after video-assisted thoracic surgery (VATS) for lung cancer. A prospective, randomized controlled trial study design is adopted to provide new strategies for rehabilitation management in patients with lung cancer. We hypothesize that remote symptom management based on ePROs can improve exercise adherence in patients with lung cancer after VATS compared with usual care.

## Methods

### Study Setting

**Figure 1:**
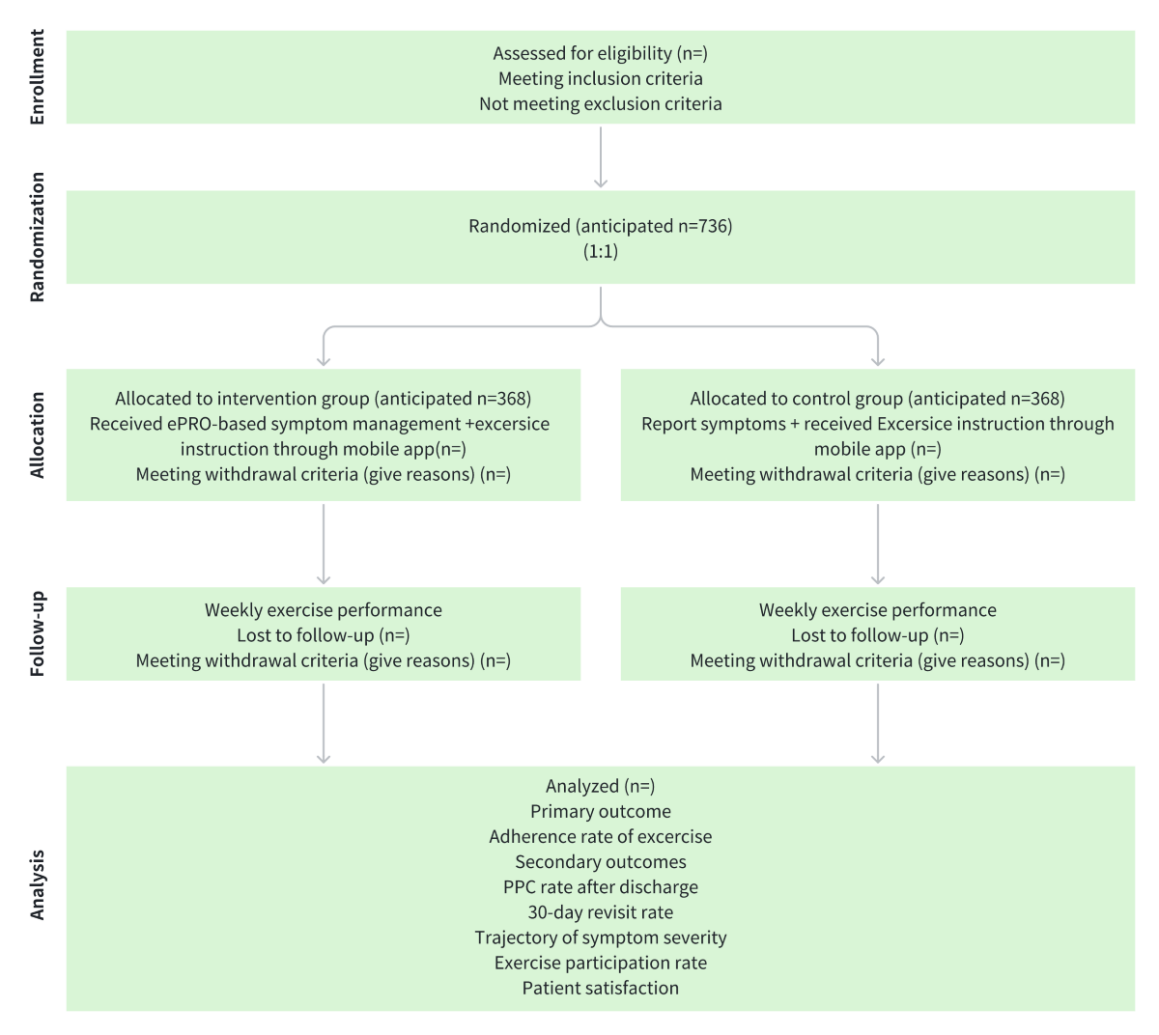
Flowchart of this parallel-group randomized controlled trial. ePRO: electronic patient-reported outcome; PPC: postdischarge pulmonary complication.

This is a single-center, prospective, superiority, randomized controlled trial, consistent with the SPIRIT (Standard Protocol Items: Recommendations for Interventional Trials) guidelines [20]. Participants will be recruited from the Department of Thoracic Surgery, Zhongshan City People’s Hospital, Guangdong, China, which performs approximately 600 lung cancer surgeries annually. The findings will be reported based on the CONSORT (Consolidated Standards of Reporting Trials) guidelines [21]. The study flowchart is shown in Figure 1. This trial has been registered on ClinicalTrials.gov (NCT05990946), where detailed information about the study protocol, inclusion and exclusion criteria, interventions, outcomes, and ethical approval can be found. The trial registration process was completed before the enrollment of the first participant.

### Eligibility Criteria

Inclusion criteria for the participants are (1) being aged 18-75 years; (2) undergoing VATS, including lobectomy or segmentectomy; (3) being able to use smart devices and completing electronic questionnaires; and (4) providing informed consent. The exclusion criteria are (1) conversions to open thoracotomy during surgery, (2) preoperative Eastern Cooperative Oncology Group score >1, (3) received neoadjuvant therapy, (4) previous thoracic surgery history, (5) unable to exercise due to physical impairments, (6) continuous systemic corticosteroid use within 1 month, (7) unresolved toxicity above grade 1, (8) significant medical history, (9) uncontrolled comorbidities, (10) postoperative length of stay >14 days, and (11) other unsuitable conditions in the investigator’s judgment.

### Participant Recruitment

Patient recruitment will commence before discharge, including preoperative patients awaiting scheduled surgery. Eligible patients will be randomly assigned before discharge after confirming they can properly operate the smartphone app.

After discharge, patients will be reminded to use the app for symptom and exercise logging. The app provides an incentive for participating in the 4-week rehabilitation program between discharge and the first postoperative clinic visit.

### Randomization and Allocation Concealment

Each potential participant will be assigned a unique 3-digit screening number in sequence. Eligible participants will be randomly allocated 1:1 to the intervention and control groups.

Randomization will be performed using a central Interactive Web Response System (IWRS), which is deployed on a third-party platform (Huawei Cloud) and was developed by the Shuyu app development team. The IWRS uses a block randomization algorithm to generate the allocation sequence and ensure balance between the treatment groups. After confirming eligibility, the study site will enter participant information into the IWRS and receive the allocation. Withdrawn participants will retain their randomization number without re-enrollment. The randomization code will be securely stored in the IWRS throughout the study.

### Blinding

Due to the nature of interventions, blinding of participants and care providers is not feasible. However, data collectors and statisticians will be blinded to group allocation. The data collectors, who are research nurses, will be trained to administer questionnaires and collect data consistently according to the study protocol. They will collect data at baseline through in-person interviews and then weekly for 4 weeks after discharge through telephone interviews.

### Interventions

Participants will use the app “Shuyu” (Module type: TH001, Developed by Shanghai CinoCore Health Technology Co) for perioperative management.

Preoperatively, nurses will instruct app use and provide education. The app will assign individualized exercises and prompt logging. Patients will complete baseline PRO measurements. The Perioperative Symptom Assessment for Lung Surgery (PSA-Lung) was used for PRO assessments. The PSA-Lung scale includes 7 symptom items (pain, coughing, shortness of breath, disturbed sleep, fatigue, drowsiness, and distress) and 2 functional items (interference of activity and walking). Each symptom’s severity was rated between 0 (the absence of symptom) and 10 (the worst imaginable symptom). Similarly, functional items were also rated on a scale between 0 (no interference) and 10 (complete interference). The PSA-Lung scale development team has verified its reliability and validity in patients who undergo lung cancer surgery, and the research results suggested adequate reliability and validity. The relevant articles have been submitted for publishing, and the preliminary results were announced at the 28th Annual Conference of the International Society for Quality-of-Life Research [22]. Permission to use the PSA-Lung scale in this study has been obtained from the scale development team.

Postoperatively during hospitalization, nurses will guide app-based rehabilitation, doctors will supervise exercises and PROs, and patients will complete daily PROs without alerts.

Before discharge, the care team will evaluate if patients can properly operate the app. Eligible patients will be randomly allocated into groups by the nurse entering the patient’s screening number and discharge date in the provider app. Based on the pregenerated randomization code from the central system, this process will automatically allocate patients into the intervention or control group, unlocking the corresponding outpatient module.

After discharge, the app continues to offer postoperative education and exercise logging, sending daily reminders at 9 AM and follow-ups at 5 PM if logs are incomplete. Weekly phone checks by staff will confirm exercise log accuracy. Noncompliance or inaccessibility after 3 phone attempts will result in protocol violation and withdrawal.

Reminders for PRO assessments are set for 9 AM on the day of discharge and on postdischarge days 3, 7, 14, 21, and 28. A follow-up reminder will be sent at 5 PM if the assessment remains uncompleted. These assessments must be completed on the specified days, and the analysis will include patients who have logged exercises but have not submitted PRO assessments. For participants who are unable to continue using the ePRO system, a manual follow-up plan will be implemented to collect PRO data through telephone or email on the day following the scheduled assessment days to avoid conflict with system reminders.

### Comparison

#### Intervention Group

The intervention group will be notified to complete the PRO measures on the designated days.

If any of the 5 core symptoms (pain, cough, shortness of breath, sleep disturbance, and fatigue) scored ≥4 in the ePRO questionnaire, alerts will be triggered on the provider end, prompting the assigned doctor and nurse to initiate remote guidance and intervention through the app through text or phone call within 24 hours following standardized procedures in the operation manual. Interventions will be based on PRO scores and include recommendations for self-management, medications, and clinic visits. In addition, patients who report any of the 5 core symptoms will immediately receive automated internet-based self-management suggestions generated by the system after submitting the PRO questionnaire.

**Figure 2:**
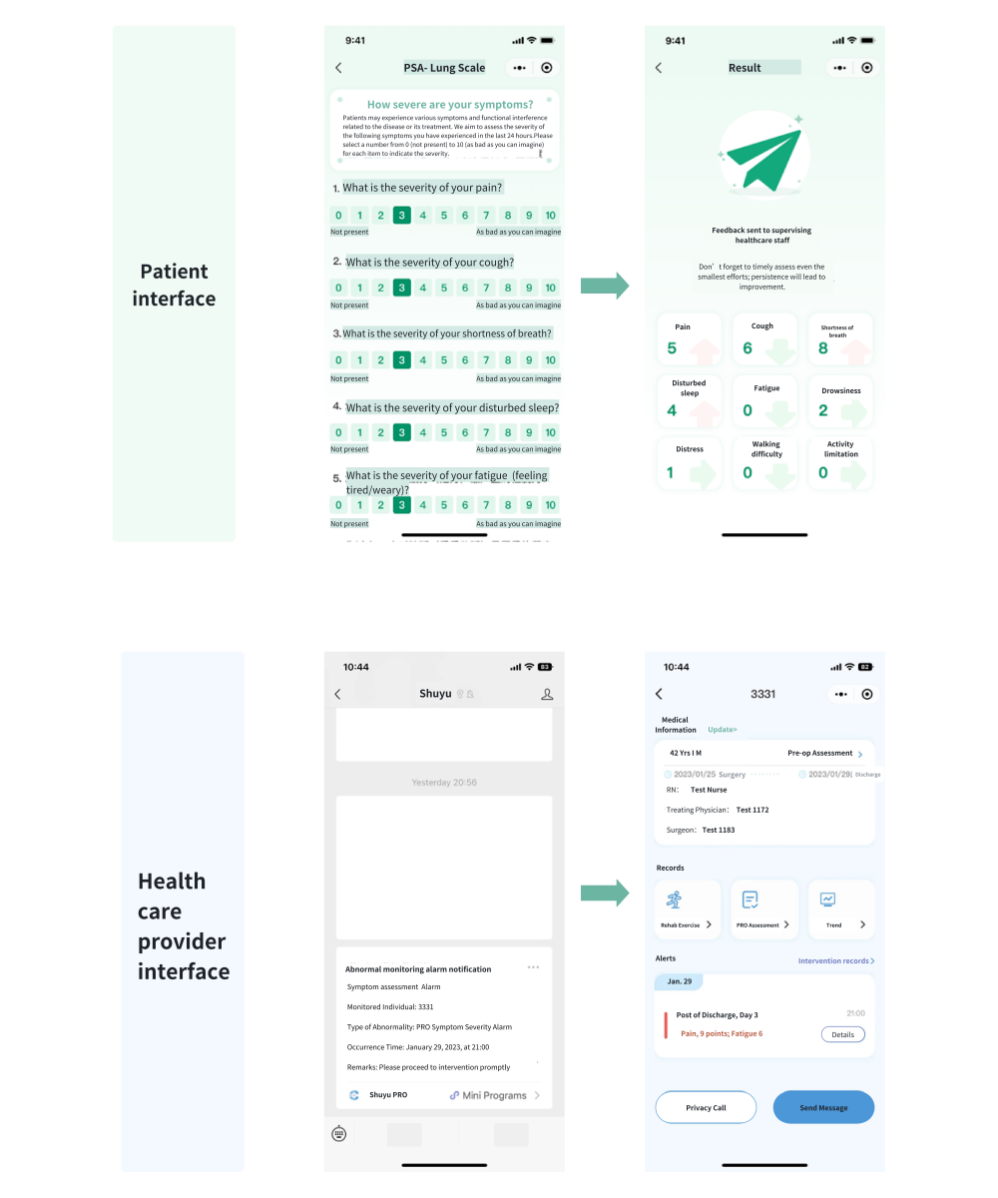
Workflow of patient self-reporting via the shuyu app and healthcare provider alert interface.

The intervention will focus on the management of these 5 core symptoms since they were identified as critical symptoms for postoperative management in previous studies on remote PRO-based symptom management after lung cancer surgery in China [16]. Based on recommendations from the National Comprehensive Cancer Network and published studies, symptom scores ≥4 are considered moderate severity or above [23,24]. Therefore, a symptom score of 4 is set as the threshold for triggering alerts. Details of the patient self-reporting process and health care provider alert interface are shown in Figure 2.

Symptom management in the intervention arm will adhere to the latest guidelines and be standardized across providers through standard operating procedure manuals, as shown in Multimedia Appendix 1. In cases of severe symptoms, physicians may advise patients to temporarily pause or adjust their exercise plans to ensure safety.

#### Control Group

The control group will complete PRO measures on the same days without triggered alerts or self-management suggestions.

All patients, regardless of group assignment, will receive guidance on seeking medical attention through conventional means for severe symptoms. Severe symptoms are defined as individual symptom scores ≥6 on the PSA-Lung scale.

#### Withdrawal Criteria

Participants meeting the following withdrawal criteria will be removed from the analysis: (1) no primary lung cancer on pathology, (2) non-microscopically complete resection, (3) stage IV disease, (4) initiation of adjuvant therapy during follow-up, (5) severe protocol violation (nonadherence to instructions, random responses, etc), and (6) voluntary withdrawal.

### Study Outcomes

#### Primary Outcome

The primary outcome is the rehabilitation exercise adherence rate over 4 weeks after discharge, defined as the proportion of patients completing the prescribed outpatient exercise regimen. Exercise completion will be ascertained based on patient self-reports through the app and verified through weekly phone follow-ups. Referencing exercise guidelines for cancer survivors from the American Cancer Society, ≥150 minutes of moderate to vigorous intensity physical activity per week (equivalent to 150 minutes of brisk walking) is considered adherent [25].

#### Secondary Outcomes

Secondary outcomes are listed in Textbox 1.

**Secondary outcomes and their measurement criteria.**

- Postdischarge pulmonary complication (PPC) rate
  Proportion of patients experiencing pulmonary complications within 30 days after discharge. Complications will be graded using the Clavien-Dindo classification [26], categorizing PPCs into 5 grades, as shown in Multimedia Appendix 2.
- 30-day revisit rate
  Proportion of nonscheduled revisits within 30 days after discharge.
- Trajectory of symptom severity
  Changes in symptom and interference scores on the electronic patient-reported outcome questionnaire from discharge to 30 days after discharge.
- Exercise participation rate
  Number of days with exercise logged within 4 weeks after discharge. Any day with exercise logged is considered participation, regardless of whether the target duration is achieved.
- Patient satisfaction: score on the 4-item questionnaire including
  Whether the app helped with rehabilitation and symptom control;
  Overall satisfaction rating;
  Whether the app caused daily life disruption; and
  Likelihood of recommending the app to others. Each item is rated from 0 to 10.

#### Other Data

The study will record time intervals from alert to provider intervention in the app backend. Questionnaires and interviews will also survey provider acceptance of the app. Participant demographic information, tumor characteristics, clinical management, treatment outcomes, adverse events, and follow-up data will be collected at different time points. All adverse events will be evaluated and managed by thoracic surgeons.

### Sample Size

The primary outcome is the rehabilitation exercise adherence rate, defined as the proportion of patients completing the prescribed exercise regimen. Adherence is defined as completing the prescribed exercise regimen, calculated from self-reported app data and verified through weekly follow-ups.

The sample size was calculated to detect a 10% difference in adherence rates between the intervention and control groups, with 80% power and a 2-sided significance level of .05. The control group adherence rate was assumed as 70% based on previous studies [27-30]. The 10% clinically important difference was determined through expert consultation, considering their practical experience and the potential impact on patient outcomes, despite limited direct literature support. The dropout rate was estimated as 20% from previous digital intervention trials (from 3.75% to 18.3%) [31-33]. Dropouts include discontinuation of app use and loss of follow-up.

Using a *z* test with a 10% proportion difference, 80% power, 5% type I error, and 20% dropout rate, the required sample size is 368 per group, 736 in total.

### Data Analysis

Statistical analyses will be performed based on both the intention-to-treat principle and the per-protocol principle. The intention-to-treat population will include all randomly assigned participants, while the per-protocol population will include participants who provide baseline PRO data and measures for at least 2 additional time points, with at least 1 calculable weekly exercise duration logged. Participants meeting withdrawal criteria will be excluded from all analyses. Statistical inferences will adopt 2-sided tests at a significance level of .05, with 2-sided 95% CI for estimation. The primary outcome, exercise adherence rate, will be compared between groups using the Pearson *χ*^2^ test or Fisher exact test. Secondary outcomes will be analyzed as follows: PPC rate and 30-day revisit rate will be compared using *χ*^2^ test or Fisher exact test; symptom severity trajectory, which involves repeated measurements, will be assessed using linear mixed effects models to account for within-subject correlation; exercise participation rate will be compared using Wilcoxon rank sum test; and patient satisfaction scores will be compared using the Student 2-tailed *t* test or Wilcoxon rank sum test. The time from alert to intervention and provider acceptance will be summarized descriptively. Baseline characteristics will be compared using 2-tailed *t* test, Wilcoxon rank sum test, *χ*^2^ test, or Fisher exact test, as appropriate. Missing data will be handled through multiple imputation or maximum likelihood estimation methods.

### Data Monitoring and Interim Analysis

A Data Safety Monitoring Board will be set up, including 1 clinical physician and 1 data manager, to conduct independent data monitoring. No interim analysis is planned considering the low-risk nature and short study duration.

### Patient and Public Involvement Statement

Patients and the public will not be involved in the design, recruitment, or implementation of this study. Study results will be disseminated to applicants. As disseminating results to participants is not standard practice in China, we have no plans for participant-directed dissemination. Study participants will be informed that final results can be accessed through our future publications.

### Ethics Considerations

The study protocol was approved by the Ethics Committee of Zhongshan City People’s Hospital in December 2022 (approval K2022-285). All recruited patients will be required to give written informed consent. Any subsequent amendments to the protocol will be submitted for further review and approval. Study findings will be disseminated through peer-reviewed publications and conference presentations.

## Results

The enrollment of study participants started in December 2023 and is expected to end in March 2025. An interim analysis is planned to assess the feasibility and preliminary effects of the intervention. The final comprehensive analysis of the results is planned for May 2025, after all data have been collected and thoroughly reviewed.

## Discussion

### Conclusions

This study primarily investigates the impact of outpatient symptom management on exercise adherence after minimally invasive lung surgery. Remote symptom monitoring based on ePROs provides an innovative approach to improve patient self-management and rehabilitation compliance. While previous digital interventions have focused on education and coaching, this study explores a new precision rehabilitation model that actively monitors patient-reported symptoms and provides timely medical feedback. Although symptom-related exercise interruptions may occur in both groups, the ePRO-based interventions are designed to help manage symptoms and provide personalized exercise guidance, which may potentially mitigate the impact of symptoms on exercise adherence.

Potential findings from this preliminary study include (1) providing initial evidence on whether ePRO-based remote symptom management can improve outpatient exercise adherence, which has been a neglected area in previous research; (2) demonstrating the feasibility and acceptability of implementing such a personalized symptom-exercise comanagement model in local patients; and (3) exploring its potential to reduce patient symptom burden and postoperative complications. The findings from this study will provide valuable insights into the barriers and facilitators of implementing ePRO-based symptom management in real-world settings, informing future efforts to expand and optimize outpatient care for postoperative patients. The planned analyses will also help elucidate the complex relationships between symptom alerts, symptom management, and exercise adherence in this context. Understanding these relationships is crucial for optimizing the design and implementation of integrated symptom-exercise management interventions for postoperative patients. If the findings support the feasibility and potential effectiveness of this innovative rehabilitation approach, it could inform future larger-scale studies and efforts to improve postoperative care for a broader patient population. The research framework and findings will contribute to advancing telehealth for patient-centered and digitally enabled care models.

### Limitations

This study also has some limitations. First, the single-center design may limit generalizability. Second, the strict inclusion or exclusion criteria may restrict the eligible population, such as excluding patients unable to use smartphones or with poorer reading comprehension. Third, the eligibility criteria may cause selection bias and limit external validity, although stringent criteria and verification of exercise data are adopted. Further pragmatic trials in more heterogeneous populations are warranted to validate broader generalizability and effectiveness. Fourth, the lack of blinding for researchers and participants may introduce bias into the results. Fifth, the short follow-up precludes evaluation of potential long-term impacts on exercise habits.

In summary, this unblinded randomized controlled trial aims primarily to provide preliminary evidence on the effects of ePRO-based symptom management on outpatient exercise behaviors, evaluating the feasibility of this management approach. Larger studies in real-world diverse populations are needed to further validate its generalizability and effectiveness.

## Appendix

Multimedia Appendix 1 Standard operating manual for symptom intervention.

Multimedia Appendix 2 Clavien-Dindo classification of surgical complications.

## Abbreviations

CONSORT: Consolidated Standards of Reporting Trials
ePRO: electronic patient-reported outcome
IWRS: Interactive Web Response System
PPC: postdischarge pulmonary complication
PSA-Lung: Perioperative Symptom Assessment for Patients Undergoing Lung Surgery
SPIRIT: Standard Protocol Items: Recommendations for Interventional Trials
VATS: video-assisted thoracic surgery

## Data Availability

The data sets generated during and/or analyzed during this study are available in the ClinicalTrials.gov repository. The protocol will be published on an open-access repository.
