# Supplementary material for: Impact of Remote Symptom Management on Exercise Adherence After Video-Assisted Thoracic Surgery for Lung Cancer in a Tertiary Hospital in China: Protocol for a Prospective Randomized Controlled Trial
Source: JMIR Res Protoc. 2025 Jan 1;14:e60420. doi: 10.2196/60420 (PMC11736221; doi:10.2196/60420)
Supplement: Multimedia Appendix 1 [file resprot_v14i1e60420_app1.pdf]

Standard operating manual for symptom intervention

Symptom Intervention Rules

Symptom	Severity	Intervention Rules
Pain	Moderate<br>(score 4-6)	Counseling + Education+ Oral NSAIDs/Opioids.
	Severe<br>(score 7-10)	Counseling + Education + Oral NSAIDs/Opioids + (topical NSAID gels applied to incision site, local nerve blocks, cold therapy, acupuncture, moxibustion, massage, cognitive behavioral therapy, etc.) ± Referral for evaluation (ask if immediate evaluation is desired).
Cough	Moderate<br>(score 4-6)	Counseling + Education + Pharmacological interventions (Antitussives - OTC cough suppressants e.g. Ambroxol oral solution/tablets, Chuanbeipipa syrup, Dextromethorphan oral solution/tablets).
	Severe<br>(score 7-10)	Counseling + Education + Pharmacological interventions (Antitussives – prescription cough suppressants e.g. Codeine tablets/oral solution) ± Referral for evaluation (ask if immediate evaluation desired).
Shortness of breath	Moderate<br>(score 4-6)	Counseling + Education + Breathing exercises + Pharmacological interventions (Bronchodilators e.g. Aminophylline oral solution/tablets, Theophylline, Montelukast).
	Severe<br>(score 7-10)	Counseling + Education + Breathing exercises + Pharmacological interventions (Bronchodilators e.g. Aminophylline oral solution/tablets, Theophylline, Montelukast) + Oxygen/Nebulization ± Referral for evaluation (ask if immediate evaluation is desired).
Sleep disturbance	Moderate<br>(score 4-6)	Counseling + Sleep hygiene education (Regular morning/afternoon exercise, increased daylight exposure, limiting electronics before bed, avoiding large meals/fluids within 3 hours of sleep, avoiding nicotine/caffeine before bed, maintaining

		dark/quiet/comfortable sleep environment, consistent sleep and wake times, no daytime naps or naps <30 minutes).
	Severe (score 7-10)	Counseling + Sleep hygiene education (same as above) + Pharmacological interventions (melatonin, sedative hypnotics, etc.) ± Referral for evaluation.
Fatigue	Moderate (score 4-6)	Counseling + Education + Non-pharmacological interventions (Physical exercise - household chores, walking, jogging, swimming, yoga, resistance training; dietary counseling).
	Severe (score 7-10)	Counseling + Education + Non-pharmacological interventions (Physical exercise - household chores, walking, jogging, swimming, yoga, resistance training; Dietary counseling; Physical therapy – massage; Psychosocial interventions – psychoeducation, cognitive behavioral therapy, supportive therapy, etc.) ± Referral for evaluation.

Symptom Intervention Operating Procedures

Symptom	Operating Procedures✻
Pain	①Explain that pain is caused by surgery and is normal, will gradually improve, and most patients do not require analgesia at discharge.<br>②Explain the importance of postoperative pain control and misconceptions: Facilitates cough, sputum expectoration and early ambulation, benefits recovery; untreated pain adversely affects deep breathing, sputum clearance and mobilization, may lead to hypoxemia, atelectasis, pneumonia, DVT etc.<br>③Explain postoperative analgesic modalities and potential side effects: Oral meds, injections, IV, PCA pump; most common side effects are constipation, dizziness.<br>④Explain non-pharmacological pain relief techniques: Local nerve blocks, cold therapy, acupuncture, moxibustion, massage, CBT, TENS.<br>⑤Assess pain severity: Teach patients to use 0-10 pain scale in PSA-Lung; objective rating at each time.<br>⑥Advise self-management techniques: Slow breathing, reading, listening to music, massage, immobilizing incision area during activity.

	⑦Risk Assessment and Management: Assess individual risk factors, monitor pain level, sedation, and respiratory status, prevent falls and constipation, and regularly review and adjust the pain management plan to ensure patient safety.
Cough	①Explain the causes of cough: Surgery-related, will gradually improve, and most patients do not require antitussives at discharge.<br>②Assess cough severity: Teach patients to properly utilize the 0-10 cough scale in PSA-Lung for objective rating at each time.<br>③Recommend self-management techniques: Avoid exposure to irritant environments.<br>④Risk Assessment and Management: Assess cough severity and characteristics, monitor for signs of respiratory distress or hemoptysis, and adjust antitussive therapy based on patient's response and potential side effects.
Shortness of breath	①Explain causes of dyspnea: Loss of lung volume from surgery; Residual chest wall trauma. Lung function usually recovers to baseline in ~3 months.<br>②Assess dyspnea severity: Teach patients to use 0-10 dyspnea scale in PSA-Lung; objective rating at each time.<br>③Advise self-management techniques: Deep breathing; Breathing exercises >6 times daily, >5 minutes each time.<br>④Risk Assessment and Management: Assess dyspnea severity and associated symptoms, monitor oxygen saturation and respiratory rate, and adjust breathing exercises and pharmacological interventions based on patient's response and potential side effects.
Sleep disturbance	①Explain causes of poor sleep: Surgery and other symptoms like pain. Will gradually improve.<br>②Assess sleep quality: Teach patients to use 0-10 sleep scale in PSA-Lung; objective rating at each time.<br>③Advise self-management techniques: Regular morning/afternoon exercise - calisthenics, brisk walking, jogging, yoga; Increased daylight exposure; Limit pre-bed electronics; Avoid large meals/fluids before bed; Avoid nicotine/caffeine before bed; Maintain dark/quiet/comfortable sleep environment; Consistent sleep/wake schedule; No daytime naps or naps <30 min.<br>④Risk Assessment and Management: Assess sleep disturbance severity and contributing factors, monitor for excessive daytime sleepiness or adverse reactions to sleep aids, and adjust sleep hygiene strategies and

	pharmacological interventions based on patient's response and potential side effects.
Fatigue	①Explain causes of fatigue: Surgery and other symptoms like pain, sleep disturbance. Will gradually improve.<br>②Assess fatigue severity: Teach patients to use 0-10 fatigue scale in PSA-Lung; objective rating at each time.<br>③Advise self-management techniques: Energy conservation (task prioritization, delegation, using equipment, postponing nonessential activities, pacing, scheduling rest, maintaining routine); Distraction (reading, music, games, socializing); Finding meaning in present circumstances (focusing on meaningful interactions, dignity).<br>④Risk Assessment and Management: Assess fatigue severity and impact on daily functioning, monitor for signs of exertional intolerance or overexertion, and adjust physical activity and supportive interventions based on patient's response and potential safety concerns.

Note: ✻Developed based on literature, expert consensus, and guidelines.
