# Supplementary material for: Impact of Remote Symptom Management on Exercise Adherence After Video-Assisted Thoracic Surgery for Lung Cancer in a Tertiary Hospital in China: Protocol for a Prospective Randomized Controlled Trial
Source: JMIR Res Protoc. 2025 Jan 1;14:e60420. doi: 10.2196/60420 (PMC11736221; doi:10.2196/60420)
Supplement: Multimedia Appendix 2 [file resprot_v14i1e60420_app2.docx]

Multimedia Appendix 2. Clavien–Dindo Classification of Surgical Complications

| **Degree** | **Definition** |
| --- | --- |
| I | Any deviation from the normal postoperative course without need of intervention beyond the administration of anti-emetics, antipyretics, analgesics, diuretics,electrolytes, and psychical therapy^a^ |
| II | Complication requiring pharmacological treatment with other medicines beyond the ones used for complications of degree I |
| III | Complications requiring surgical, endoscopic, or radiological intervention |
| III-a | Intervention without general anesthesia |
| III-b | Intervention under general anesthesia |
| IV | Life-threatening complication requiring admission tointensive care unit |
| IV-a | Uniorgan dysfunction (including dialysis) |
| IV-b | Multiorgan dysfunction |
| V | Death |

a This degree also includes wound infections opened at the bedside.
